# Integration of Blockchain, IoT and Machine Learning for Multistage Quality Control and Enhancing Security in Smart Manufacturing

**DOI:** 10.3390/s21041467

**Published:** 2021-02-20

**Authors:** Zeinab Shahbazi, Yung-Cheol Byun

**Affiliations:** Department of Computer Engineering, Jeju National University, Jejusi 63243, Korea; zeinab.sh@jejunu.ac.kr

**Keywords:** blockchain technology, quality control, machine learning, security, big data, internet of things

## Abstract

Smart manufacturing systems are growing based on the various requests for predicting the reliability and quality of equipment. Many machine learning techniques are being examined to that end. Another issue which considers an important part of industry is data security and management. To overcome the problems mentioned above, we applied the integrated methods of blockchain and machine learning to secure system transactions and handle a dataset to overcome the fake dataset. To manage and analyze the collected dataset, big data techniques were used. The blockchain system was implemented in the private Hyperledger Fabric platform. Similarly, the fault diagnosis prediction aspect was evaluated based on the hybrid prediction technique. The system’s quality control was evaluated based on non-linear machine learning techniques, which modeled that complex environment and found the true positive rate of the system’s quality control approach.

## 1. Introduction

Generally, the smart manufacturing system movement in recent years has been moving toward integrating blockchain technology, physics, and cyber capabilities to capture their advantages, and toward using detailed information to expand system-wide flexibility and compatibility [1,2]. It is often regarded as Industry 4.0—a term originated in the German government project to encourage the 4th generation of manufacturing using the concept of cyber-physical systems, equipment, and processes to create easy decision making in smart factories [3,4,5]. Smart manufacturing leverage correlates with the advanced data velocity, volume, and variety connected with big data. Applying big data techniques increases the strength of analyses and assists with predictive analysis [6]. The mentioned capabilities come up in most industries but with various factors and speeds, i.e., their needs, based on suppliers and installation methods. Consequently, the common ground that exists between multiple industries can help to explain and improve the capabilities of specific industries [7,8].

**Figure 1:**
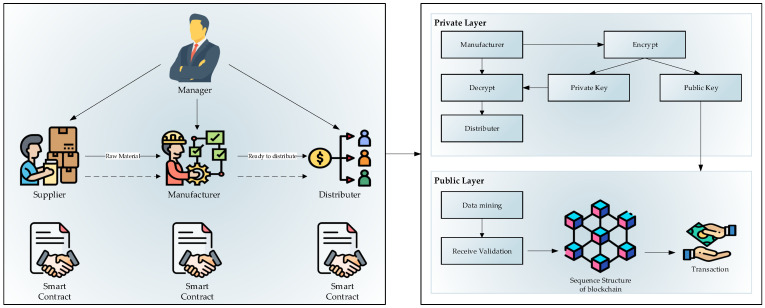
Overview of the proposed system.

In recent years, blockchain technology’s growth and desirability have gathered attention, especially in the financial industry [9,10,11]. Most of the regular blockchain applications are based on asset transfers and distributing information across networks based on smart contracts, which are considered ideal for business operations and industry sectors. The main focus of smart manufacturing in Industry 4.0 is to qualify the relationship between different manufacturing units, facilities, retailers, etc., for further support manufacturing industries, based on the total manufacturing value chain [12]. This process affects automating and optimizing the operations, improving flexibility, safety, cost reduction, productivity, and profitability. However, Industry 4.0 comes with many advantages and challenges in the manufacturing sector that stand in the way of the benefits. Most of the challenges are summarized in connectivity—exchanging information among various machines, etc. [13]. Figure 1 shows the overview of the process. This scenario considers the transaction process between the manufacturer and the distributor. There are two main layers in the proposed blockchain in this system to perform the manufacturer’s transaction: public and private layers. In the first step, the manager is in contact with the supplier, manufacturer, and distributer to handle manufacturing and control the situation to decrease the fault rate during the process. There are set rules in smart contracts for the manufacturing process. Each layer contains the information which is stored in the blockchain. The private layer focuses on the manufacturer’s product distribution process. In the private layer, the manufacturer first decrypts the provided data and then distributes them. Similarly, the dataset is encrypted into two main parts: the public key and the private key. The private key is directly associated with the decrypted file and the public key with the public layer. The public layer is focused on the transaction section of this procedure. The first step is the data mining process to manage the transaction dataset and receive the validation. Next is storing the dataset in the sequence structure of the blockchain, and finally, the transaction is validated.

The main contributions of this paper are as follows:

- Real-time monitoring based on the IoT environmental sensors.
- Reducing the latency of decision making using blockchain.
- Applying blockchain to secure the decentralized and transparent transactions.
- The use of smart contracts to enhance the manufacturing network.
- Predictive analysis based on the fault diagnosis of the manufacturing system.
- Applying big data techniques to manage the massive manufacturing dataset.

This remainder of the paper is divided as follows: Section 2 presents the practical literature review of the current industrial and technological processes. Section 3 presents the proposed manufacturing model system’s architecture. Section 4 presents the system’s performance and results, and we conclude this paper in the conclusion section.

## 2. Related Work

This section encompasses details about smart manufacturing and identifies some recent industry issues overlooked by the scientific community.

### 2.1. Big Data Challenges and Revolution in Smart Manufacturing

As the development of industry in the second decade of the new millennium progressed, analytics’ next-generation also started improving. The first step toward progress was increased device complexity, which needed revolutions in manufacturing procedures; e.g., we design 3D devices now rather than 2D, and new devices such as FinFET [14] were conceived [15,16]. The second step was lead by the new market drivers, which were toward lower power but faster and smaller devices. Internet of Things (IoT) technology is applied to these devices. This technique is used to configure the devices, which are connected over the Internet. Data collection and analysis from different means—feedback, production, enterprise, requests, etc.—improved the decision making process in smart manufacturing [17,18]. During these developments, manufacturers and customers provided feedback and their points of view related to the products, which helped the manufacturers improve product quality, design, etc. Big data analysis helps a manufacturer identify customer preferences and identify the failures of a product in real-time, which improves the potential of data-driven marketing for predictive smart manufacturing [19,20,21].

Some big data technology can handle processing and storing the massive volumes of data in the manufacturing industry, e.g., NoSQL MongoDB, Apache Kafka, and Apache Storm. Apache Kafka is a scalable messaging system suitable for making real-time applications [22]. The advantages of this system are the high-throughput, scalablity, and fault-tolerance. Some research showed positive outcomes when applying it to healthcare, sensor data generated based on IoT technology, etc. Alfin et. al. [23] presented real-time data monitoring in healthcare. The proposed system’s applied techniques are a combination of Apache Kafka and MongoDB to save the patients’ data extracted from sensors. In [24], a car parking system based on cloud technology was proposed, which contains the Apache Kafka technique. The system can manage a large amount of sensor data related to increases in the number of clients.

### 2.2. Blockchain Technology in Smart Manufacturing

Blockchain technology is revolutionary for data security, data transmission, fault tolerance, and transparency [25]. The distributed ledger is key to this process. A blockchain is a security-focused structure with excellent potential, efficient transparency, and decentralization. This technology became public through Bitcoin [26], and researchers extended this technology with various applications and in different fields. Nowadays, the applications of blockchain are not limited to cryptocurrency and are applied in many other areas, e.g., agriculture [27], education [28], healthcare [29,30,31,32,33,34,35,36], finance [37], transportation [38,39], and supply chains [40,41,42,43,44,45,46,47,48,49,50]. In [51], the authors developed the blockchain-based agriculture supply chain management and traceability system. The main goal of this system is to trace the food products [52] and manage the supply chain. The IoT-based agricultural supply chain system uses two separate platforms in the blockchain network, Ethereum and Hyperledger Fabric. These two networks differ in various aspects, e.g., transaction mode and latency. The benefit of applying this system is being sure that the stored information is secure. In [53], the authors focused on secure access to data based on digital tools integrated into a chain. The main solution for that goal is blockchain technology, which distributes the digital information without giving copy permission and manages the time-stamp dataset in the network used to connect the services and the system. The implementation of this approach is based on the permissioned blockchain.

Cloud manufacturing is another type of manufacturing system which is based on customer-driven manufacturing. The proposed system’s main focus is using distributed resources as a service and making them capable of providing cyber-physical manufacturing control based on manufacturing as a service. The cloud architecture is centralized, creating problems of trust and security for the user and the service [54]. In [55], public and private networks were used as manufacturing service providers in the cloud. At the service provider level, a public blockchain was used, and at the level of the workshop, a private blockchain was used. The data were collected based on the level of the machine. In [56], a cloud-based blockchain network was proposed to enable trust in the network without a trusted moderator. However, manufacturers wished to reveal certain information, but the proposed model lacks the ability to authorize such sharing. Thus, the efficiency of operation and the quality of service were inadequate [57]. Table 1 presents blockchain technology’s challenges and opportunities in a few related research areas.

### 2.3. Machine Learning Technologies in Smart Manufacturing

Based on the modern technologies used for machine learning—IoT, big data, etc.—in smart manufacturing, the industry’s main focus is creating an intelligent manufacturing environment. The modern manufacturing system contains various sensors for collection of data in the different formats and structures. The sensor data come from product lines, the equipment, activities, sensors of environmental conditions, etc. This section’s main topics are the analysis of large volumes of data and real-time processing [76]. Machine learning (ML) and artificial intelligence (AI) contain various tools and techniques for quality control and improving production processes, pattern identification, and the roles of automatic learning from datasets [77]. ML and AI contain several opportunities for data aggregation and generating standard process insights, e.g., preventive maintenance, forecasting the production, quality control, etc. The predictive maintenance deals with data to develop schemes for identifying anomalies. Forecasting the production based on the trends can leverage overtime to accurately estimate the productivity cycle. Quality control inspection applications can use various ML techniques to generate dependable results without human intervention. Similarly, it is significant for manufacturing workers to adopt standards and protocols in open communication. In [78], the main focus was customer satisfaction in the manufacturing system’s production model. The integration of AI and information communication enabled the manufacturing standard to be high and customized the factory based on optimizing the operations, intelligent decision making, self-perception, etc.

## 3. Design and Architecture of Blockchain-Based Smart Manufacturing Quality Control

**Figure 2:**
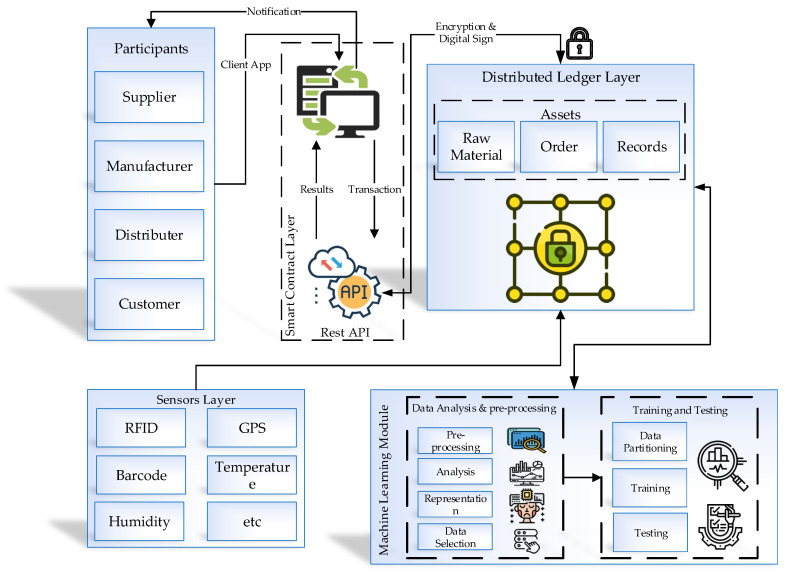
System architecture diagram.

In this section, the detailed architecture of the proposed system is discussed. Figure 2 illustrates the proposed system’s architecture, which is based on blockchain-based quality control. The proposed system contains four main layers, an IoT sensor layer, a distributed ledger layer, a smart contract layer, and a business layer with the various functions. Blockchain technology safely distributes the ledger for assessing quality, assets, logistics, and transaction information. The defined smart contract provides the intelligence, privacy protection, and automation in the presented system, and IoT sensors extract the real-time data. The machine learning modules applied in this process are for pre-processing and analyzing data.

The first layer, the sensor layer, uses GPS to trace the products’ logistics and location information. RFID gives the transaction, quality, and asset information. Due to the high costs of RFID, barcodes can utilize processes when the accuracy standards are not required and data are few. Furthermore, other sensors can be used to collect related information—temperature, humidity, etc. The second layer is the distributed ledger layer, which contains four main blockchain aspects: transactions, assets, logistics, and quality data. All enterprises in the supply chain keep copies of this data—the supplier, the manufacturer, the logistics manager, the retailer, and the financial institution operator. This information is used to perform quality control and ensure the efficiency of the system. The third layer is the smart contract layer, which is used to improve supply chain efficiency by gathering and sharing data. To avoid the privacy issues, digital identities are used for controlling the authority to access the data. The reason for using this process is that competitive enterprises in the same supply chain will need to keep some information confidential. Finally, the business layer contains the different business activities. Similarly, it is able to manage and control the quality and support contracts via blockchain.

### 3.1. Real-Time Quality Control

**Figure 3:**
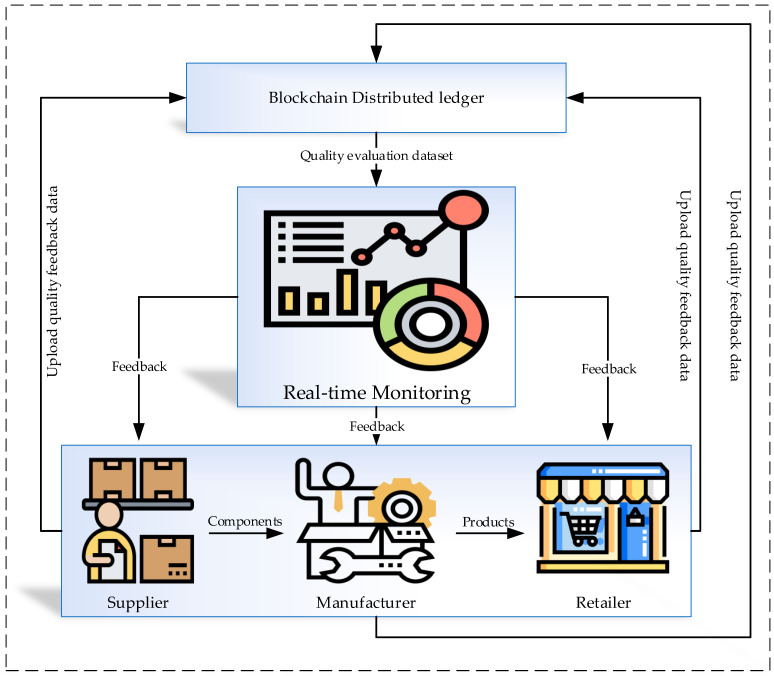
Real-time controlling and quality monitoring.

The increasing number of companies and factories in the world is making the effectiveness of blockchain technology greater. The companies involving machinery, networks, participants, parts, products, and logistics all face security problems when sharing their datasets inside and outside the factory. Blockchain’s best place in any industry is based on the manufacturer managing to identify its needs and problems correctly. By providing challenges, opportunities, and understanding of the industry, the manufacturer can select the best option while mitigating the issues of blockchain technology. Transparency and trust in blockchain technology are important at every stage of production, from gathering raw materials to delivering the final product. Some of the key points are monitoring the supply chain for better transparency, monitoring the sources of materials, managing company identities, tracking the assets, securing the quality, and the adoption of standards. Figure 3 illustrates real-time data monitoring in the process of production using a blockchain system. The real-time data quality and product quality processing are evaluated based on smart contracts, and the feedback of this process is sent to the supplier, manufacturer, etc. The system can provide different suppliers smart contracts using digital identities. Each component has its own digital identity with a specific authentication code for the blockchain. Furthermore, a manufacturer cannot read this information, which is the case to avoid revealing the data to other suppliers. Manufacturers are able to control the means of monitoring though, based on the smart contracts’ rules.

### 3.2. Digital Identity

**Figure 4:**
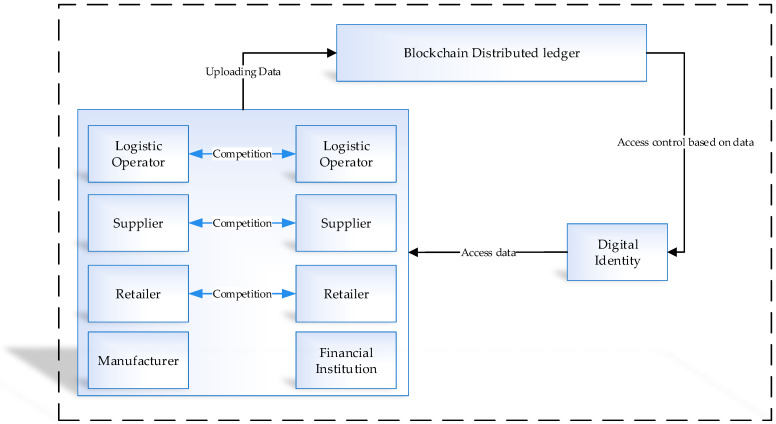
Distributed ledger digital identity based on blockchain.

Digital identity has a great role in measuring system security in interconnected devices. To use the online services, a user might need to make a profile on various websites and use his personal information to be available for using services. This information is stored without the user knowing, and it is accessible to third parties. By applying the decentralized service of blockchain for online activities, each user can have a separate digital ID based on these IDs, and with the digital watermarking techniques, the user’s transactions can be executed. Based on this process, the user data can be stored in the permission network, and they are accessible only for the user with the right to access it. Figure 4 presents the blockchain distributed ledger based on the digital identity. The data collected from the logistics operators, suppliers, retailers, manufacturers, and financial institutions uploaded in the distributed ledger and access control to this data are based on digital identity.

### 3.3. Contract Automation and Logistics Planning

**Figure 5:**
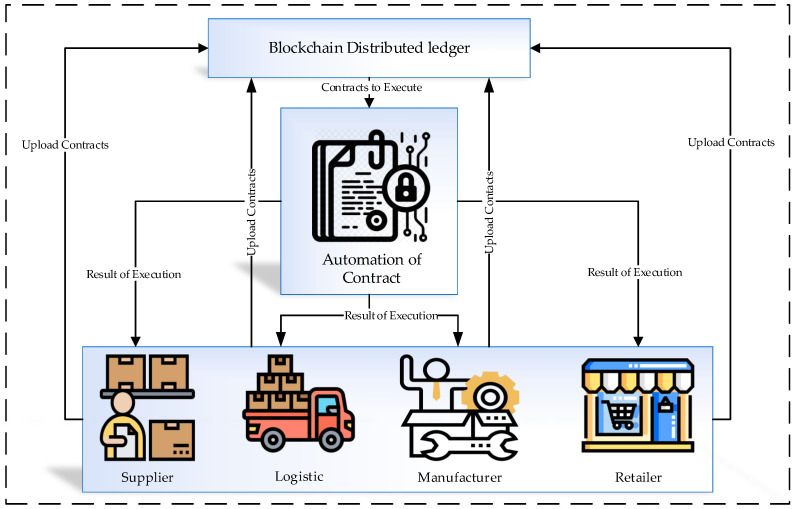
Distributed ledger contract automation based on blockchain.

From among the data in the blockchain and the smart contracts, suppliers are available to access customer feedback and analysis related to products to help them improve their production. Data collected based on the IoT sensors regarding environmental information—temperature, humidity, etc.—are used to trace and train the product’s transport. The usefulness of smart contracts in the logistics system is to route the product’s transportation intelligently. The transportation information is accessible for manufacturers and suppliers. The logistics plans in a smart contract are defined based on product position and quantity. Simultaneously, the digital identity supports the system to keep it confidential for logistics providers and competition. Figure 5 presents the contract automation in the distributed ledger of the blockchain. The output of contract execution is able to be accessed by the supplier, logistics manager, manufacturer, and retailer. Similarly, they also upload the contracts in the distributed ledger. The uploaded contracts are the input for contract automation, and the system follows this process to improve the security and defined rules.

### 3.4. Blockchain Transaction Execution Process

**Figure 6:**
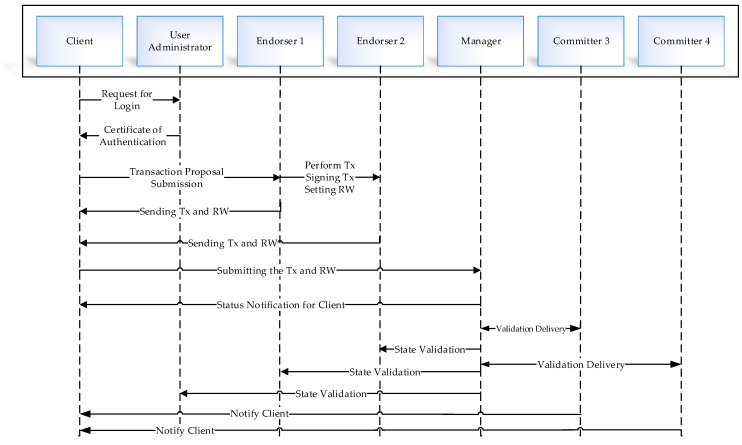
Procedure of transaction execution based on blockchain technology.

In this section, the transactional process in the manufacturing industry is explained. Step by step transaction accomplishment is illustrated in Figure 6. The user’s ability to connect to the blockchain system is based on the front-end application using his registered ID. User registration is the administrator’s responsibility to allow certain users to accomplish the right transactions. A transaction proposal needs the user’s login information and a transaction request submission based on the registered documents. The transaction records share with the nodes after completing the process. There are two types of nodes: endorser and committer nodes. Endorser peers are responsible for performing the transaction request and validating it, and otherwise rejecting it. The committer pairs first, authorizes the transaction, and writes the transaction into the ledger block. The endorser is a particular type of committer used to hold smart contracts. Moreover, the endorser is applied to extract the selected transaction’s smart contract before upgrading the ledger in its simulated environment to receive the transaction proposal. The endorser simulated environment is a RW set. The RW set includes the relevant data from before the transaction, and it performs the transaction in a simulated environment. In the next step, the signed transaction is returned to the client based on the RW set, and the client sends it back to the manager for transaction delivery—at this stage it has been updated with the RW set to order the dataset toward a block. The data are compared with the real-world transaction information, which is done by nodes, and after matching, the contract is written into the ledger. Finally, updating the ledger is based on the data provided. At last, the committer node sends the submitted notification to the client for the state validation. The process between the client and the blockchain network uses the REST API.

### 3.5. Machine Learning-Based Predictive Analysis

**Figure 7:**
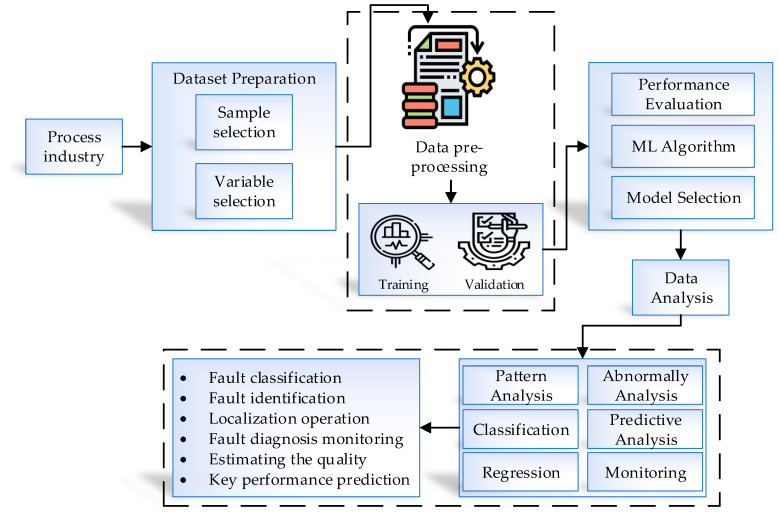
Machine learning fault detection process.

During the past few decades, machine learning has become part of the industrial process to predict and make easier decision making for further steps and developments. Data extraction is based on the useful information and identifying the new patterns and predictions, and making the data samples far more simplified. Consequently, the decision making can be faster than before. All this has a direct effect on the manufacturing life cycle. The main goal of applying machine learning techniques is to supply a new perspective to the the manufacturing industry. Figure 7 presents the process of fault detection in the proposed system. The data preparation is an important and essential step in machine learning to extract the necessary and related datasets, examine the structure, and select the direction and samples. To extract the essential dataset and utilize it, changes in conditions and operations also need to be identified. To improve the quality of a dataset, data pre-processing and transmission are required. After preparing the training dataset, the machine learning algorithm that meets our needs for modeling must be selected. By selecting a model, the necessary parameters are specified. In the next step, the performance evaluation is completed. There are many techniques with which to evaluate the performance and validate the model—cross validation, parameter sensitivity analysis, model stability analysis, etc. After completing the whole process of modeling the dataset and validating the performance, data analysis techniques are applied to further improve our approach. Some analysis techniques include clustering, monitoring, fault diagnosis, fault classification, and quality monitoring. The fault diagnosis is done to give detailed information related to a fault found during the process. Depending on the fault diagnosis method, the fault’s main cause might be in the process or related to a special sensor. After clearing the fault diagnosis output, the performance evaluation report is generated. Soft sensing or prediction techniques are able to evaluate the key performance of the procedure. The predictive data models can extract and add to the online prediction process based on the ordinary variables’ relationship. The result shows the real-time prediction output based on the regression and prediction models.

## 4. The Implementation Process of the Purposed Smart Manufacturing Method

In this section, the results and implementation process of the proposed integrated method are evaluated. Table 2 presents the tools and technologies applied in the implementation and the required configuration. The operating system on which the process was implemented and run was Ubuntu Linux 18.04.1 LTS using an Intel(R) CPU Core(TM), i7-8700, at 3.20 GHz. The blockchain environment needed the docker engine version 18.06.1-ce, and the docker composer version suitable for this process was 1.13.0. We have used the open-source Hyperledger Fabric V1.2 blockchain technology, and the primary memory used was 32 GB. The programming language was Python via tensor-flow with the Composer-Playground IDE platform, and the CLI (command line interface) tool used was the Composer REST Server, which is a famous tool used for deploying most composers.

### 4.1. The Smart Contact Development of the Case Study in Smart Manufacturing

**Figure 8:**
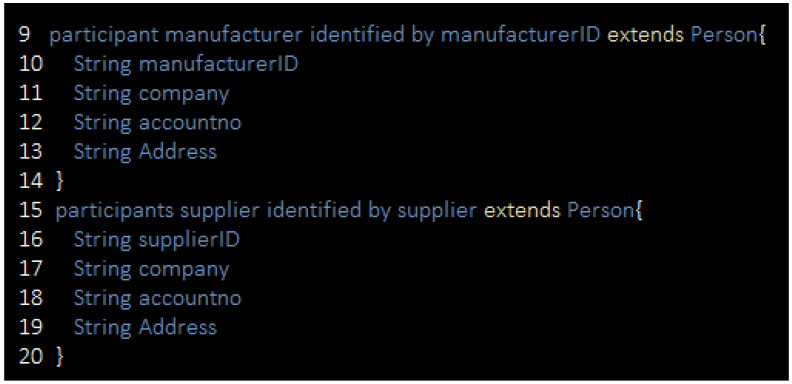
Definition of Hyperledger composer participants.

Our development based on blockchain requires a suitable environment, for which Hyperledger is a good option. One of the important aspects is the design of a smart contract for the business network. A smart contract on the basis of Hyperledger contains four main components that define the participants, the business logic script, and the rules of access control to secure the access point of the database. The business network is run based on the participants and their assets, which can accomplish the transaction. The participants in this system are suppliers, manufacturers, distributors, and retailers. Exclusively, the assets in this system are set as raw materials, orders, and records. The participants are presented in the business network in Figure 8. A transaction based on a smart contract involves users’ interactions with assets, performance of the transaction, a private network for the participants, and every other activity during the smart contract’s development. The event functions are also considered as part of the transaction and the execution process of the same transaction. Table 3 presents the events and transactions of our procedure.

### 4.2. The Distributed Ledger in the Manufacturing System

**Figure 9:**
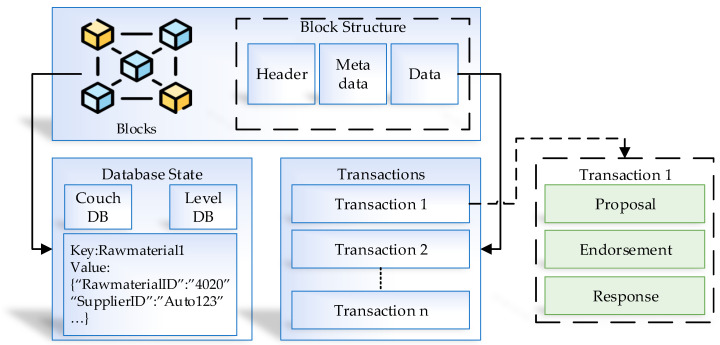
Ledger communication and the block transaction process.

The main basis of Hyperledger can be summarized as two sections. The first is a blockchain, and the second is the world-state. Another ability of Hyperledger is to configure the world-state databases to receive access in ledger phases based on the current set of values. World-state is capable of automatically storing a value and checking it without the need for fully logging information. The key value contains the world-state data and a reference. The ledger communication and block transactions are presented in Figure 9. The updates and changes are automatically done with the world-state database. Couch DB and Level DB are the world-state suitable options. Level DB is the default state to save the smart contract information in the existing and paired nodes through the network. The Couch DB is the answer for rich query environments and is modeled in the smart contract. Instead of saving the key values, the Couch DB stores the actual data. The results and information from REST API are supported in Couch DB. Based on these advantages, the Couch DB is used in the proposed environment.

## 5. Results and Discussion

In this section, the predictive analysis of machine learning algorithms, evaluation metrics, blockchain execution results, and smart manufacturing vision is presented in detail. The experimental settings of the blockchain environment and machine learning approach are included.

### 5.1. Machine Learning-Based Predictive Analysis

**Figure 10:**
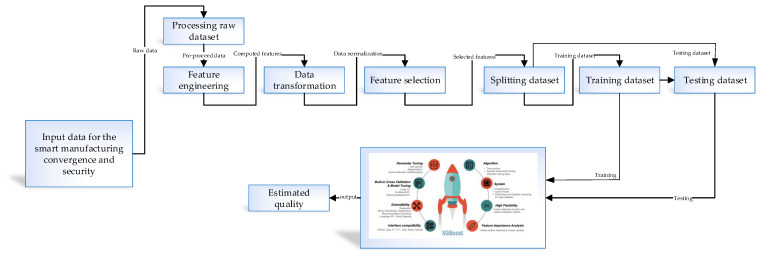
Architectural diagram of the predictive analysis based on XGBoost.

Implementing the predictive analysis in this procedure followed the Python language and scikit-learn module for the eXtreme Gradient Boosting (XGBoost) model, which is open-source technology. XGBoost is the basis of eXtreme Gradient Boosting for optimization, and it is a flexible and highly efficient algorithm for non-linear and numeric datasets. It avoids the overfitting problem due to processing. Figure 10 presents the architectural diagram of the predictive analysis in this approach. There are three main sections in the prediction process. The first section is the data collection process, done in the manufacturing environment. The second section is the data processing—to make the data suitable and prepare them for further industry processes. The last section is applying the XGBoost prediction algorithm to evaluate and predict the proposed system’s quality in the manufacturing environment. The data pre-processing section contains seven main steps: processing the raw data, feature engineering, comparing the features based on data transformation, normalizing the dataset, selecting the features, splitting the dataset into training and test sets, and finally applying the XGBoost algorithm.

Equation (1) evaluates the XGBoost objective function:

(1) $$\lambda^{t}=\sum_{i=1}^{m}A\left(z_{i},{\hat{z}}_{i}\hat{\left(t-1\right)}+D_{t}\left(R_{i}\right)\right)+\Phi \left(D_{t}\right)$$

In this evaluation process, $z_{i}$ is the training dataset’s real value. *A* is the learning function, and *t* is the number of iterations. $D_{t}$ is the function for every iteration, and $R_{i}$ represents the function of the combination of all features in every iteration.

#### Evaluation Metrics

To evaluate how well the system identifies or excludes the binary classifier, accuracy is used as a statistical measure. Equation (2) evaluated the accuracy of this procedure. $X_{a}$, $X_{b}$, $Y_{a}$, and $Y_{b}$ represent the true positive, true negative, false positive, and false negative. A high impact accuracy demonstrates good performance.

(2) $$Accuracy=\frac{X_{a}+X_{b}}{X_{a}+X_{b}+Y_{a}+Y_{b}}$$

Equation (3) evaluates the recall of this procedure. The recall shows the correctly identified true positive proportion, which is the best metric for model selection.

(3) $$Recall=\frac{X_{a}}{X_{a}+Y_{b}}$$

Equation (4) evaluates the precision based on the correct and positive values identified. Similarly, it measures the cost in false-positive conditions.

(4) $$Precision=\frac{X_{a}}{X_{a}+Y_{a}}$$

**Figure 11:**
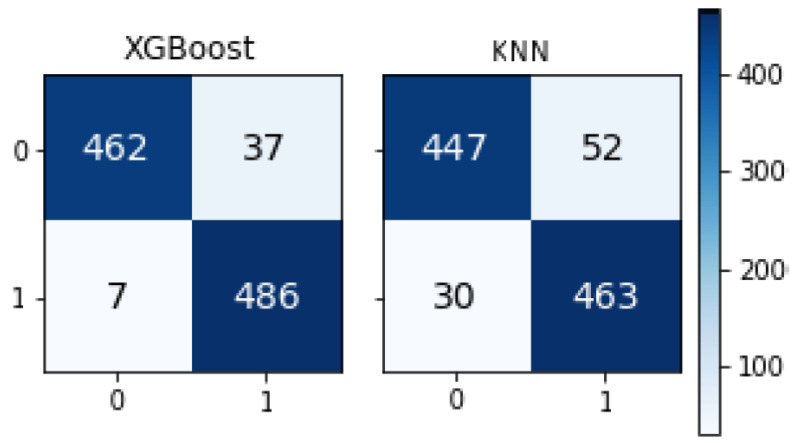
Confusion matrices of machine learning algorithms.

Figure 11 shows the three high-performance machine learning algorithms’ confusion matrices in the presented system. XGBoost had the highest output.

**Figure 12:**
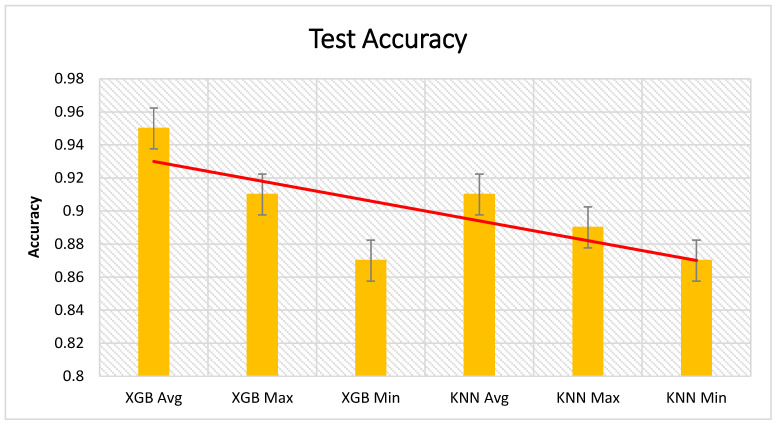
Cross validation of the test accuracy results.

**Figure 13:**
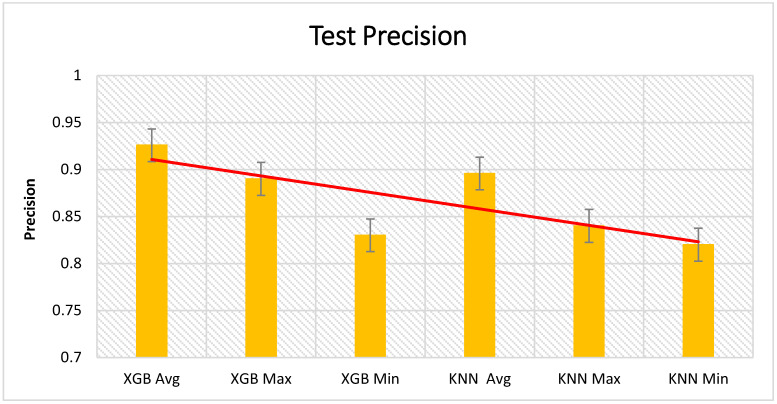
Cross validation of the test precision results.

**Figure 14:**
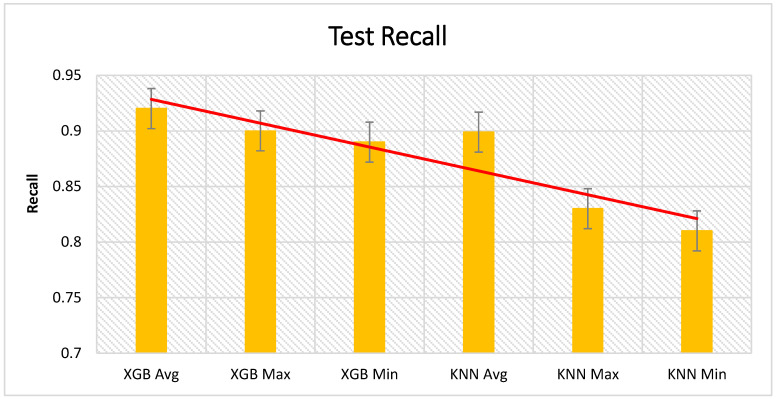
Cross validation of the test recall results.

Figure 12, Figure 13 and Figure 14 present the test results from the cross validation, which are divided into accuracy, precision, and recall of XGBoost and KNN algorithms. XGBoost had the highest impact in the presented approach. We have compared XGBoost with other machine learning algorithms presented in Table 4.

### 5.2. Execution Results of the Blockchain Environment

**Figure 15:**
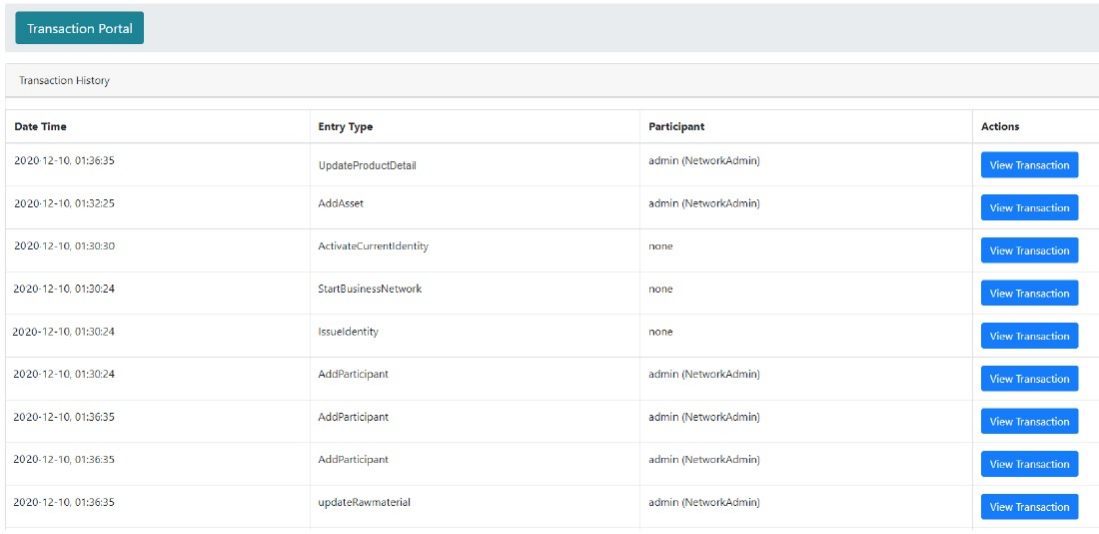
Transaction portal history information.

The blockchain network in a smart manufacturing industry is presented. In the smart manufacturing management system, the manufacturer can add, update, and delete the product details in the blockchain network. The manufacturer is supposed to fill out the web form and all blockchain network entries for adding new details. Similarly, users can update their information in the blockchain network by sending an update request in the blockchain network’s user interface. Figure 15 presents the blockchain network’s transaction history portal in the proposed system. In this portal, the transaction history and all the activities completed are related to the transactions provided. The information contains the dates, times, entry types, participants, and actions. Similarly, the transaction log file details of the network are also provided.

**Figure 16:**
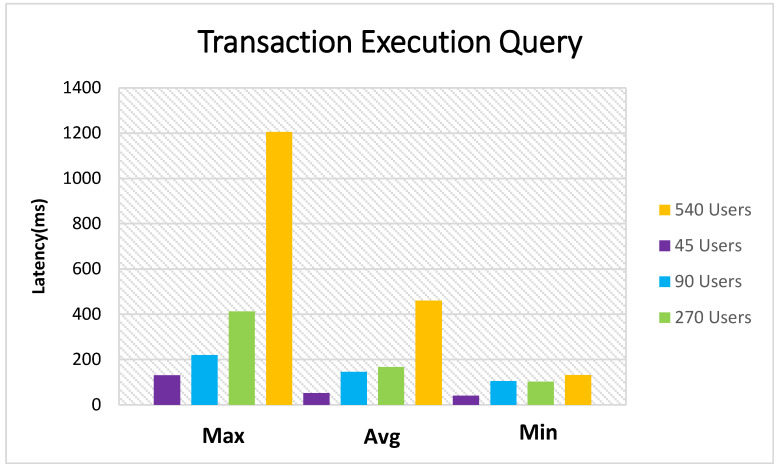
Query transaction latency.

Figure 16 presents the maximum, minimum, and average transaction latency of the process. There are various user groups with different numbers of members compared. The 540 user process had an average latency of 450 (ms); the 270 one had an average latency of 167 (ms), and the 90 user process had a 145 (ms) average latency. Finally, the 45 users had a 52 (ms) average latency in this procedure.

**Figure 17:**
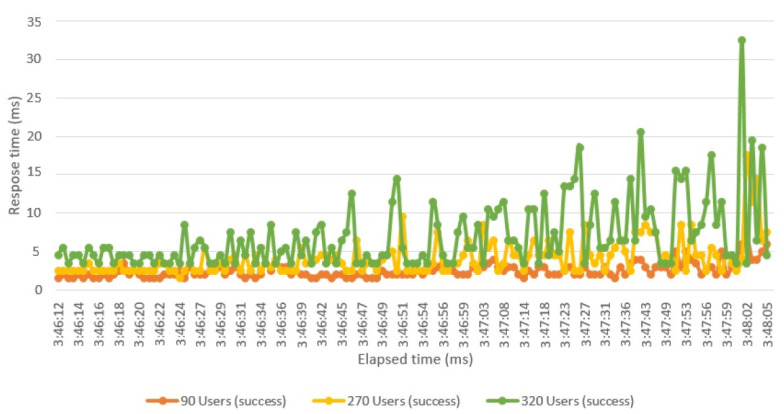
Different request response times.

Figure 17 illustrates the various response times in the network based on users’ growth. The system’s performance was evaluated based on three groups of users with 90, 270, and 320 members in the first, second, and third testing periods. There were no changes in the system’s response time in the first and second tests, but the third group’s test results contained slight changes in the system’s response.

## 6. Conclusions and Future Work

In this research, multistage quality control was evaluated based on various machine learning and blockchain-based solutions. Data validation was performed based on the performance of the classification output. A comparison between XGBoost and other ML algorithms showed that XGBoost can extract the complex relationship of the dataset and provide a quality evaluation with the highest accuracy. The presented system’s main goal—and novelty—was implementing a blockchain integrated with machine learning to improve smart manufacturing procedures and environment quality; it provided excellent results. This system offers a secure environment for manufacturers and users to improve business environments with more safety and trust. As future work, we plan to increase the network size to test and validate the system’s performance for more complicated manufacturing environments in terms of accuracy, machine learning models, etc.

## Figures and Tables

**Table 1:** Related studies of blockchain’s challenges and opportunities.

Author	Theoretical Approach	Technology Approach	Model Approach	Blockchain Application Context
Ali et. al. [58]	Systematic Literature	Blockchain	-	-
Li et. al. [59]	Cross case study, enterprises framework	Blockchain and edge Computing	-	Smart contract
Kouhizadeh et. al [60]	Decision making evaluation	Blockchain	theories of force and field in TOE framework	-
Siegfried et. al. [61]	Descriptive literature, analysis of systematic fit	Blockchain	-	-
Sun et. al. [62]	Review	Blockchain	-	Bitcoin
Easley et. al [63]	Review	Blockchain	Game theoretic	Bitcoin
Sheth et. al. [64]	Theory of contract	Blockchain	Demand supply based on economic model	Ethereum
Treiblmaier et. al. [65]	Theory building framework	Blockchain	Principal agent theory	Blockchain applications
Wang et. al. [66]	Review	Blockchain	Analysis of transaction costs	Blockchain applications
Pazaitis et. al. [67]	Case study framework	Blockchain	Decentralized cooperation	Technological solution
Jianchao et. al. [68]	Use Case	Blockchain	Five force model	-
Morkunas et. al. [69]	Firm business	Blockchain	Assets tracking in Hyperledger	Computing the power of processing
Biswas et. al. [70]	Industry	Blockchain	Transactional risk	Proof transaction records
Kumar et. al. [71]	Food Industry	Blockchain	Supply chain management	-
Kamble et. al. [72]	Sustainable supply chain	Blockchain	Foreign exchange automate payment mechanism	Privacy in different concerns
O’Leary et. al. [73]	Supply chain and accounting	Blockchain	Stock exchanges	Network design, scalability
Kurpjuweit et. al. [74]	Additive supply chain	Blockchain	Upgrade the manufacturing results	Lack of technical skills
Lin et al. [75]	Industrial internet of things	IIoT	Adaptability, confidentiality	Trust with the third part

**Table 2:** Proposed system tools and techniques of implementation.

System Component	Description
Operating System	Ubuntu Linux 18.04.1 LTS
CPU	Intel(R) Core(TM) i7-8700@3.20 GHz
Hyperledger Fabric	V1.2
Docker Engine	Version 18.06.1-ce
CLI Tool	Composer REST Server
Docker Composer	Version 1.13.0
Primary Memory	32 GB
Programming Language	Python, tensor-flow
IDE Platform	Composer-Playground

**Table 3:** Definitions of transactions in the proposed system.

Component Name	Component Type
Update Product Details	Transaction
Update Raw Material	Transaction
Update the Status of order	Transaction
Share Product Record with Wholesalers	Transaction
Share Product Record with Distributer	Transaction
Share Product Record with Notification Messages	Event
Updating the status of order	Event
Sharing the notification of order place	Event
Sharing the notification of confirmed orders	Event
Share the notification of order detail with distributers	Event

**Table 4:** Comparison results of machine learning algorithms.

Model	Training (s)	Prediction (s)	Accuracy
XGBoost	1.412	1.118	95.56
KNN	1.119	1.482	91.73
SVC	2.187	1.581	80.2
Naive Bayes	1.115	1.112	68.5
Logistic Regression	1.184	1.112	60.4

## Data Availability

No data available.
